# miRWalk: An online resource for prediction of microRNA binding sites

**DOI:** 10.1371/journal.pone.0206239

**Published:** 2018-10-18

**Authors:** Carsten Sticht, Carolina De La Torre, Alisha Parveen, Norbert Gretz

**Affiliations:** Medical Research Center, Medical Faculty Mannheim, University of Heidelberg, Mannheim, Germany; Ohio State University, UNITED STATES

## Abstract

miRWalk is an open-source platform providing an intuitive interface that generates predicted and validated miRNA-binding sites of known genes of human, mouse, rat, dog and cow. The core of miRWalk is the miRNA target site prediction with the random-forest-based approach software TarPmiR searching the complete transcript sequence including the 5’-UTR, CDS and 3’-UTR. Moreover, it integrates results other databases with predicted and validated miRNA-target interactions. The focus is set on a modular design and extensibility as well as a fast update cycle. The database is available using Python, MySQL and HTML/Javascript Database URL: http://mirwalk.umm.uni-heidelberg.de.

## Introduction

MicroRNAs (miRNAs) are small, non-coding RNA molecules of 21–25 nucleotides base length. They are involved in gene expression regulation by alignment with target gene, resulting in cleavage or repression of the target genes at post-transcriptional level [1]. They play important regulatory roles in many biological processes, including differentiation, metabolism, development and cellular signaling. Thus, identification of gene targets is important for functional characterization of miRNAs and gives new insights into biological processes that could leads to biomarkers and predictors of drug response for disease. Processes for identification and validations of microRNA targets in laboratory are mostly time consuming and expensive. These limitations have led to the development of sophisticated computational approaches of microRNA target predictions allows for narrowing down the potential targets for experimental validation.

Several computational methods to identify target genes have already been developed. Some methods rely on the conservation of binding sites (e.g. TargetScan) [2], other relies on site accessibility and thermodynamic properties to filter the seed binding sites (e.g. miRanda) [2]. Prediction algorithms use a combination of different features to increase their accuracy and compensate for the limitations of the individual features. However, there is still need of accurate with high sensitivity computational approach needed to overcome the problem generated by traditional based algorithm. Machine learning based algorithms rely on parameterization of biological data and other predicted features and are growing new era in genomics. This technique used by many prediction algorithm that generate more accurately validated miRNA-target interaction (for e.g., TarpmiR, miRGen++, MBSTAR) [3–5].

Based on prediction accuracy algorithm and the fact that most of the prediction databases were not updated for some years, we have decided to launch state of the art learning based technique with new features and transfer to miRWalk repository to an another server on a new framework to increase the accuracy and sensitvity, which allows exhaustive use of other application in this study.

## Implementation

### Data retrieval

All mRNA sequences and other necessary information (e.g. EntrezID, mRNA and CDS length, gene location and definition) of all known genes of human, mouse, rat, cow and dog were extracted from NCBI database. miRNA sequences and other information (e.g. Sanger name, MIID, genomic location of miRNA, stem loop sequence and other accession numbers like stem loop, and mature sequence) were downloaded from miRBase (version 21) [6].

TargetScan (conserved site context scores, version 7.1), miRDB (release 5.0) and the validated information from miRTarBase (version 7.0) [7] datasets were also incorporated into miRWalk framework. These platforms were chosen on the basis of their popularity and accuracy in prediction of interaction.

### Execution

Target prediction was then performed with the TarPmiR algorithm, which was developed by analyzing high-throughput expression profiling data in a random forest framework (mirdb9). With updated genomic data and the TarPmiR algorithm, we have performed genome-wide miRNA target prediction for all known transcripts (including all isoforms) from five species––human, mouse, rat, dog and cow. Data prediction was performed on a high performance computing cluster bwHPC (Baden-Wurttemberg High Performance Cluster). All the target prediction data as well as the associated genomic annotations were imported into a backend MySQL database for web presentation. The users can search for precompiled results via miRWalk web interface, using either miRNA or gene target search terms. Notably, the users have the flexibility of searching a single miRNA/gene target, or a combination of multiple miRNAs/gene targets.

### Website implementation

The miRWalk website was implemented by using the Python Django web framework running on top of a MySQL database. The D3.js Javascript library was used for visualizations and interactive features of interaction. The system is deployed on the heiCloud Platform with 16G RAM and 8-core processor CPUs. The performance of network visualization is dependent on the user’s browser. miRWalk has been tested with major modern browsers such as Google Chrome (60+), Mozilla Firefox (50+) and Microsoft Internet Explorer (10+). For a better experience, we recommend users to access miRWalk using the latest Firefox or Chrome browser from a computer with at least 4G RAM and 1280 × 800 screen resolution.

### Database update

The database has been and is updated twice a year. For this purpose, special scripts were written in Python 3, which automatically download all necessary data and files, process them and save them in the appropriate formats and tables. The actual prediction of gene miRNA interactions with TarPmiR (the most time-consuming part) is then performed on a grid server and the results are finally integrated into miRWalk database. Thus the complete database is updated every 6 months.

## Web interface

### Search single gene / miRNA

Users can give single input of miRNA ID (e.g. hsa-miR-214-3p) or Accession numbers (e.g. MIMAT0000271) based on current version of miRBase by selecting species. While searching single miRNAs, also short names or family miRNA (e.g. let-7) belonging to several miRNAs are also acceptable. In case of mRNA, users can use following ID to search interaction information of input: Gene symbols (e.g. GAS2), EntrezIDs (e.g. 10608), Ensembl-IDs (e.g. ENSG00000148935 or ENST00000454584) and RefseqIDs (e.g. NM_001143830) and click on search option to execute the query input.

### Search a set of genes / miRNAs

The Target Mining provides an advanced search option for several miRNAs or gene targets. Users may upload miRNA or gene list. When searching for miRNA gene targets interactions, full mature miRNA names are required. For the search of miRNA regulators, you may provide either NCBI gene IDs or official gene symbols.

#### Search output

**Fig 1:**
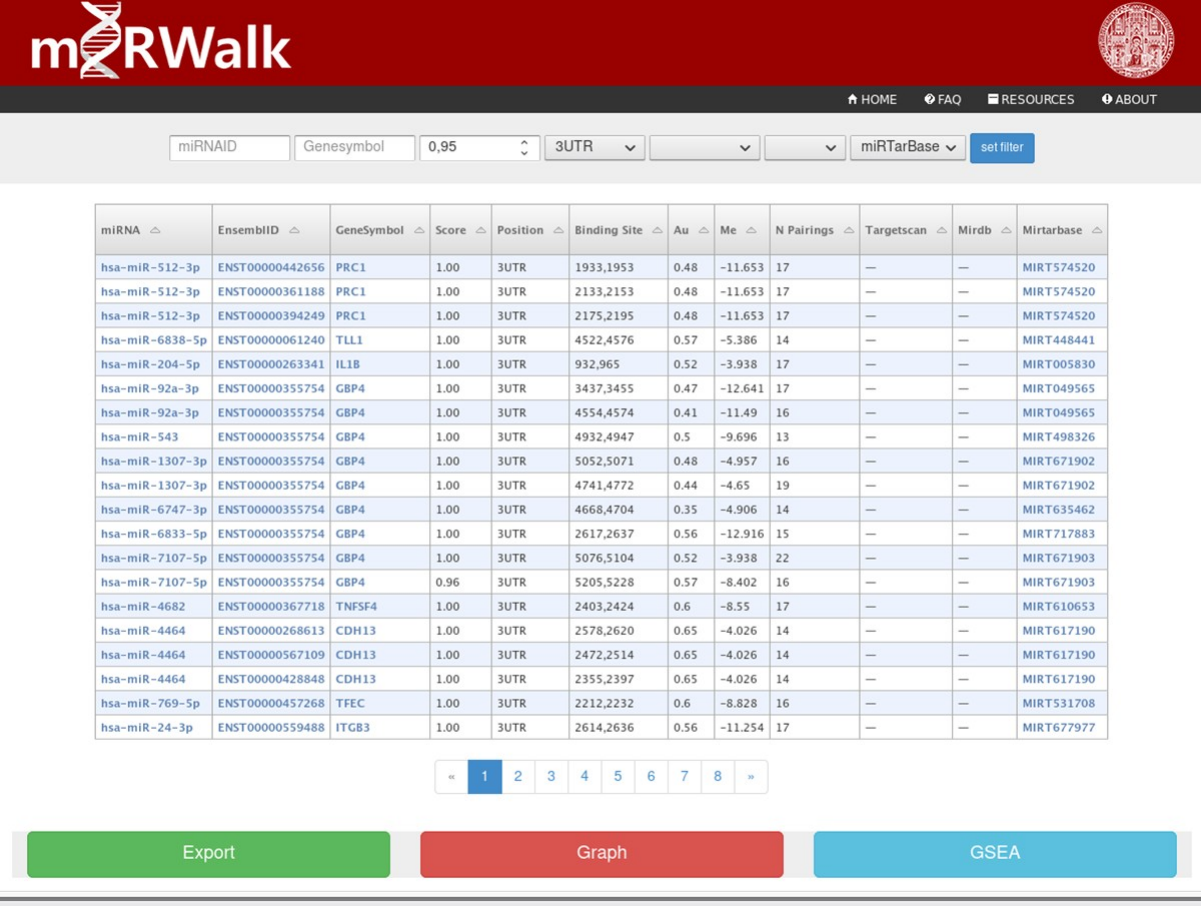
Overview of query output. Overview of results retrieved after querying several target genes. Several filter options can be set to refine the query output. The table output consists several links to other databases: miRBase (miRNA-IDs), Ensemble (Ensembl Transcript IDs) and NCBI (Genesymbols).

After searching for target interactions, different options are available for filtering the output data generated (Fig 1):

- miRNA-ID or GeneID: they show only the interactions from this miRNA or gene generated in initial columns. Users can choose Ensembl-ID (e.g. ENSG) or official gene symbols.
- Score: adjust it to filter out all results with a minimum of binding probability along with binding position (3UTR, CDS, 5UTR) with a single entry. The **score** is calculated from a random-forest based approach by executing TarPmiR algorithm for miRNA target site prediction.
- Other popular algorithms, such as miRDB or Target Scan are available to compare with the results. For only validated results, users can choose miRTarBase as filter option.

#### Export data

Users can download output of query search in a plain text format (comma separated format (.csv)). The interaction feature list calculated with TarPmiR is saved into the exported table.

#### View graph node

**Fig 2:**
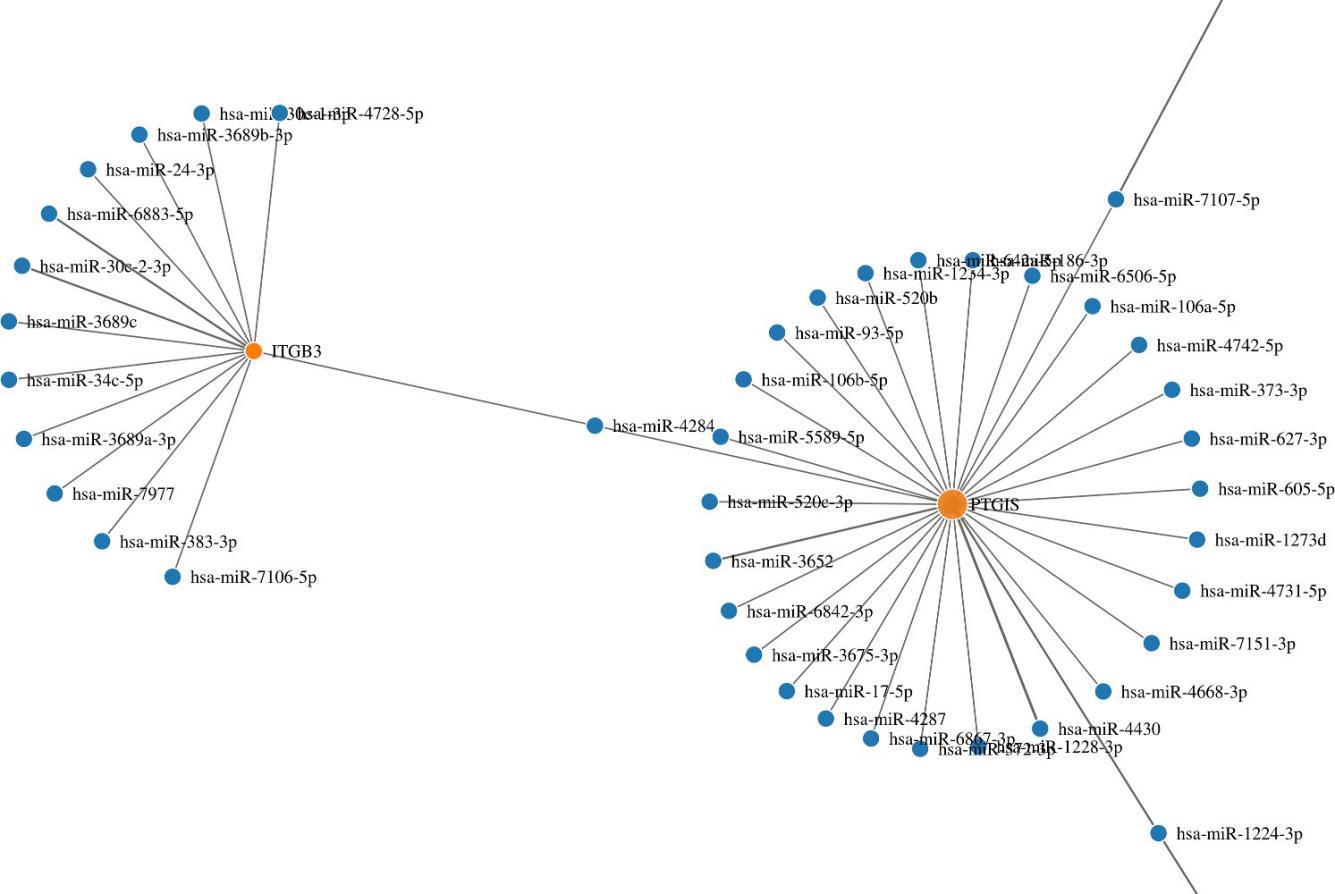
Network graph plot. The miRNA-target gene interaction can be displayed as a node graph. The graph can be exported in SVG, PNG or PDF format.

The miRNA-target gene interaction can be displayed as a node graph produced with the javascript library d3.js (Fig 2). For big networks, we recommend a powerful computer, since the graphs are calculated on client side. We have limited the number of nodes up to 10,000.

#### GSEA

The gene set enrichment analysis (GSEA) is to test whether any functional group of genes (e.g. pathways, target of a transcription factor) from the user selected list are significantly enriched among those genes of interest. miRWalk offers a standard enrichment analysis based on the hyper geometric tests (chi-square selection algorithm).

## Discussion

Many computational techniques have been developed to predict miRNA-target genes and multiple features are being introduced to help identify their target genes, such as complementarity of different regions on miRNAs, binding site conservation or target sites accessibility. Different predictive algorithms are based on different features; therefore, integrating diverse algorithms may improve target prediction. Our strategy for improving miRWalk [8] database was including prediction results of several different algorithms to cover all these factors and getting better accuracy in predicting of miRNA target gene interactions. For that, implementation of TarPmiR was of great importance since it applies a random-forest based learning approach to integrate most of these features to predict miRNA target sites and besides, it offers the possibility to extend the binding class and include new features.

## Conclusion

The miRWalk database provides up-to-date information on gene miRNA interactions. With a clearly structured and intuitive interface, users can quickly and successfully capture data, perform statistical analyses, and visualize and download Gene-miRNA networks. The free availability and the persistent updating of the data is an enormously important factor, especially in science. miRWalk (version 1) started in 2011 and is constantly being updated and further developed. This integrative approach allows users to easily identify important miRNA targets to better understand the roles of multiple miRNAs and optimize their gene targets.
